# Apigenin Inhibits the Progression of Osteoarthritis by Mediating Macrophage Polarization

**DOI:** 10.3390/molecules28072915

**Published:** 2023-03-24

**Authors:** Xueyan Ji, Wei Du, Wenqing Che, Liping Wang, Lu Zhao

**Affiliations:** 1 Jiangsu Key Laboratory of New Drug Research and Clinical Pharmacy, Xuzhou Medical University, Xuzhou 221004, China; 13206479793@163.com (X.J.); 13635654234@163.com (W.C.); wlp869861@163.com (L.W.); 2 Department of Pharmacy, The Affiliated Changzhou No. 2 People’s Hospital of Nanjing Medical University, Changzhou 213164, China; 13615136011@163.com

**Keywords:** osteoarthritis (OA), apigenin, macrophage polarization, TRPM7, MAPK

## Abstract

Objective: The overall purpose of this study was to investigate the mechanism of macrophage polarization on chondrocyte injury in osteoarthritis and the protective effect of apigenin on chondrocytes in osteoarthritis. Method: Primary chondrocytes were isolated from the knee cartilage of three-day-old mice, and cells positive for Alsine blue staining and type II collagen immunocytochemical staining were identified and used in followup experiments. Transwell coculture was performed. Chondrocytes were inoculated in the inferior compartment, and macrophages were inoculated in the upper compartment. The experimental groups were the N group, LPS group, and LPS+ apigenin group. The effect of macrophage polarization on chondrocyte inflammation and the protective effect of apigenin on chondrocytes were verified by the drug administration. Real-time quantitative PCR (qPCR) and Western blot were used to detect the expression of RNA and protein. Experimental OA was induced by modified Hulth surgery in mice. Modified Hulth surgery was performed on the mouse’s right knee to induce experimental osteoarthritis in mice, with the nonoperative right knee serving as an ipsilateral control. The mice were randomly assigned to three groups (six mice per group): the sham group, the modified Hulth group, and the modified Hulth + apigenin group. Animals were given gavage for four weeks. The protective effect of apigenin on articular cartilage was verified by histological staining and immunohistochemical analysis. Results: Histological staining showed that apigenin had a protective effect on cartilage degeneration induced by modified Hulth surgery. The PCR results showed that apigenin significantly reduced the expression levels of IL-1, IL-6, MMP3, and MMP13 in the articular cartilage of OA mice, and it had a protective effect on articular cartilage. Apigenin reduced the levels of IL-1, IL-6, TNF-α, and IL-12 in macrophages and increased the levels of MG-L1, MG-L2, ARG-1, and IL-10, which can inhibit the M1 polarization of macrophages and promote M2 polarization. In the coculture system, apigenin decreased the protein levels of TRPM7, P-mTOR, BAX, and c-caspase3 in macrophages, while significantly increasing the protein levels of Bcl2. The levels of IL-1, IL-6, MMP13, TNF-α, P38, JNK, and ERK phosphorylation were reduced in chondrocytes. Conclusion: Apigenin alleviates cartilage injury in OA mice induced by modified Hulth. Apigenin inhibits chondrocyte inflammation through the MAPK pathway. Apigenin alleviates macrophage-polarization-induced inflammatory response and chondrocyte apoptosis in the macrophage–chondrocyte coculture system through the TRPM7-mTOR pathway.

## 1. Introduction

Osteoarthritis (OA)—a progressive and degenerative disease, and the most common form of arthritis—is a leading cause of musculoskeletal pain, disability, and socioeconomic loss worldwide [1]. The pathogenesis of OA is characterized by extracellular matrix (ECM) damage and chondrocyte death [2]. Chondrocytes preserve the maintenance of articular cartilage by regulating articular cartilage structure and function, as well as ECM turnover [3]. However, the current medicinal therapy for OA is largely palliative. The most frequently used therapeutic class of drugs to treat OA is nonsteroidal anti-inflammatory drugs, which are associated with serious cardiovascular and digestive side effects [4]. Chondrocyte apoptosis is a functionally important phenomenon under physiological conditions. However, chondrocyte apoptosis is an essential pathogenic factor in cartilage degeneration and a potential target for the therapeutic prevention of OA during the progression of the degenerative process [5].

Macrophages have substantial plasticity, heterogeneity, and pluripotency, and they adapt and respond to various micro-environmental stimuli. Generally, macrophages consist of two polarization states: M1-polarized macrophages are involved in the initiation of inflammation, while M2-polarized macrophages are involved in inflammation inhibition [6]. Macrophages are polarized into M1 or M2 depending on the local immunological milieu [7]. Macrophage polarization plays an important role in the development of various diseases, including metabolic diseases such as diabetes and obesity [8], pathogen infections, asthma, tumors, osteoporosis [9], and OA [10]. As various immune cells play important roles in OA, inflammatory mediators released by infiltrating immune cells promote extracellular matrix degradation [11]. Thus, modulating inflammation by reprogramming macrophages has been regarded as an effective therapeutic strategy for the treatment of OA.

Apigenin (4′,5,7-trihydroxyflavone) is a natural flavonoid found in abundance in vegetables (including onions, parsley, and celery), in plant-based beverages (such as beer, wine, and tea), and in several kinds of herbs (including chamomile, thyme, oregano, and basil) [12,13]. It has been previously demonstrated that apigenin has a wide range of biological activities, mainly including antitumor, antidiabetic, antidepression, and antianxiety activities. Apigenin also has a potential protective effect against bone degenerative diseases. The anti-inflammatory properties of apigenin involve p38/MAPK, PI3K/Akt, and reducing the activation of the NF-κB pathway and COX-independent pathways [14]. Previous research proved that apigenin-glycoside-rich extract reduced inflammation and exerted chondroprotective effects in a rat model [15,16]. Apigenin at 30 mg kg^−1^ body weight significantly inhibited osteoclastogenesis in vivo and prevented trabecular bone loss in the femurs of ovariectomy-induced bone-loss mice [17]. In one clinical study, chamomile oil, rich in flavonoids such as apigenin 7-O-glucoside, significantly relieved knee pain in an osteoarthritis patient [18]. Moreover, it attenuated the inflammatory responses of LPS-stimulated RAW 264.7 macrophages [19]. Although apigenin has potential antiosteoarthritis effects, despite these studies, the mechanism of its protective effects on OA is still not fully understood. Therefore, the primary aim of the present study was to investigate the chondroprotective effect of apigenin on a chondrocyte/macrophage coculture system by repolarizing macrophages.

## 2. Results

### 2.1. Protective Effect of Apigenin on Articular Cartilage in Modified Hulth Surgically Induced OA Mice

**Figure 1:**
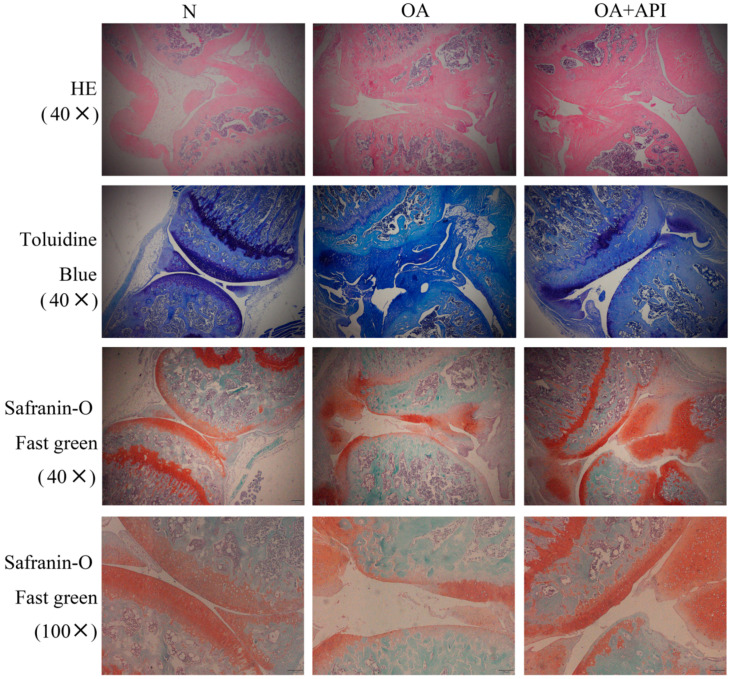
Apigenin protected articular cartilage in OA mice. Representative photographs of sections of articular cartilage with H&E, toluidine blue, and Safranin-O Fast green staining in the N, OA, and OA + API groups (magnification, ×40, ×100). Compared with the N group, the OA group exhibited serious degradation of the cartilage, obvious proteoglycan loss, and deep cartilage erosion. In contrast, the OA + API group exhibited a smoother cartilage surface and reduced the loss of proteoglycan.

**Figure 2:**
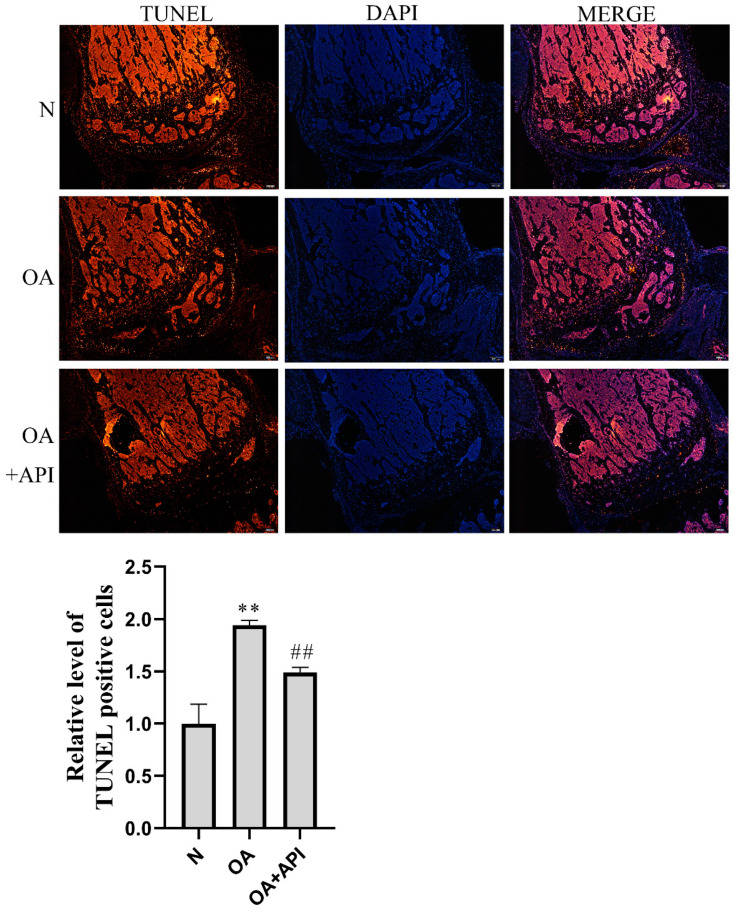
Apigenin reduced chondrocyte apoptosis in OA mice. Above are representative photographs of TUNEL staining in articular cartilage sections (magnification, ×40), The blue fluorescence is DAPI and the red fluorescence is TUNEL. Compared with the N group, the OA group showed a significant increase in TUNEL-positive cells, and the apoptosis of cells was increased. Apigenin, however, reduced apoptosis. The figure below shows the quantified fluorescence intensity of TUNEL-positive cells: ** *p* < 0.01 versus normal group (N); ## *p* < 0.01 versus OA group.

To investigate the protective effect of apigenin on OA, we first established a surgically induced knee OA mouse model. H&E, Safranin-O Fast green, and toluidine blue staining were employed for the histological evaluation (Figure 1). We observed that, compared with normal mice, OA mice presented serious degradation of the cartilage, obvious proteoglycan loss, and deep cartilage erosion. In contrast, the mice in the modified Hulth + apigenin group exhibited a smoother cartilage surface and reduced loss of proteoglycan. These results demonstrate that apigenin exerts protective effects against modified-Hulth-induced cartilage degeneration. In addition, TUNEL assay results showed more apoptotic cells in the cartilage of OA mice, while cell apoptosis was decreased in the cartilage of mice in the modified Hulth + apigenin group (Figure 2).

**Figure 3:**
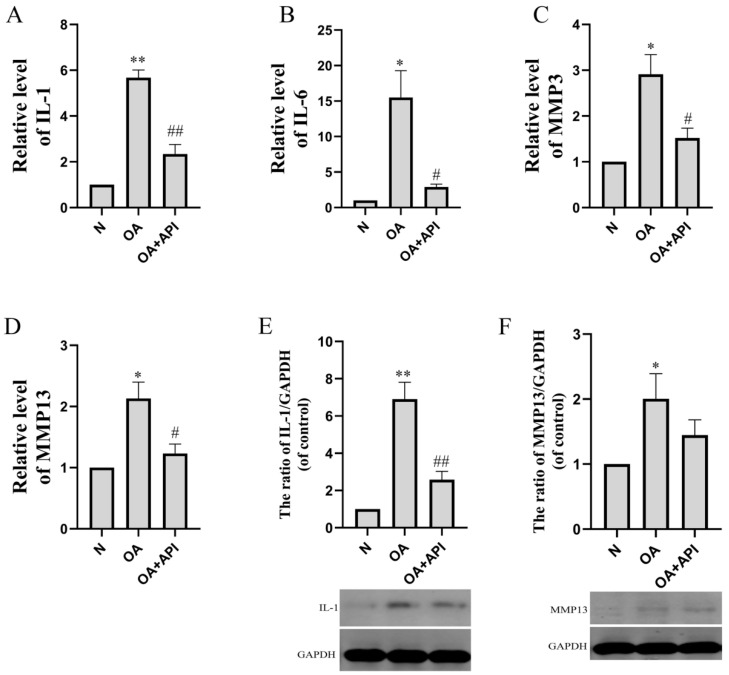
The effect of apigenin on the levels of inflammation-related mRNA and proteins of chondrocytes in OA mice. Relative quantities of inflammation-related genes are expressed as fold differences compared to the control. (**A**) IL-1, (**B**) IL-6, (**C**) MMP3, (**D**) MMP13, (**E**) IL-1 protein level, and (**F**) MMP13 protein level. Above, densitometric analysis; below, representative images of immunoblots. Data are the mean ± SD of the mean (n = 3). * *p* < 0.05 versus normal group (N); ** *p* < 0.01 versus normal group (N); # *p* < 0.05 versus OA group; ## *p* < 0.01 versus OA group.

The reverse transcription (RT)-PCR results showed that the levels of IL-1 (*p* < 0.01), IL-6 (*p* < 0.05), MMP3 (*p* < 0.05), and MMP13 (*p* < 0.05) were markedly elevated in the articular cartilage of OA mice. The expression levels of IL-1, IL-6, MMP3, and MMP13 were significantly reduced by apigenin (Figure 3). Western blot analyses showed similar results (Figure 3E,F). IL-1 and MMP13 protein levels were significantly increased in the OA group, while apigenin reduced IL-1 (*p* < 0.01) and MMP13 protein levels.

### 2.2. Apigenin Inhibits Macrophage M1 Polarization in a Macrophage–Chondrocyte Coculture System

**Figure 4:**
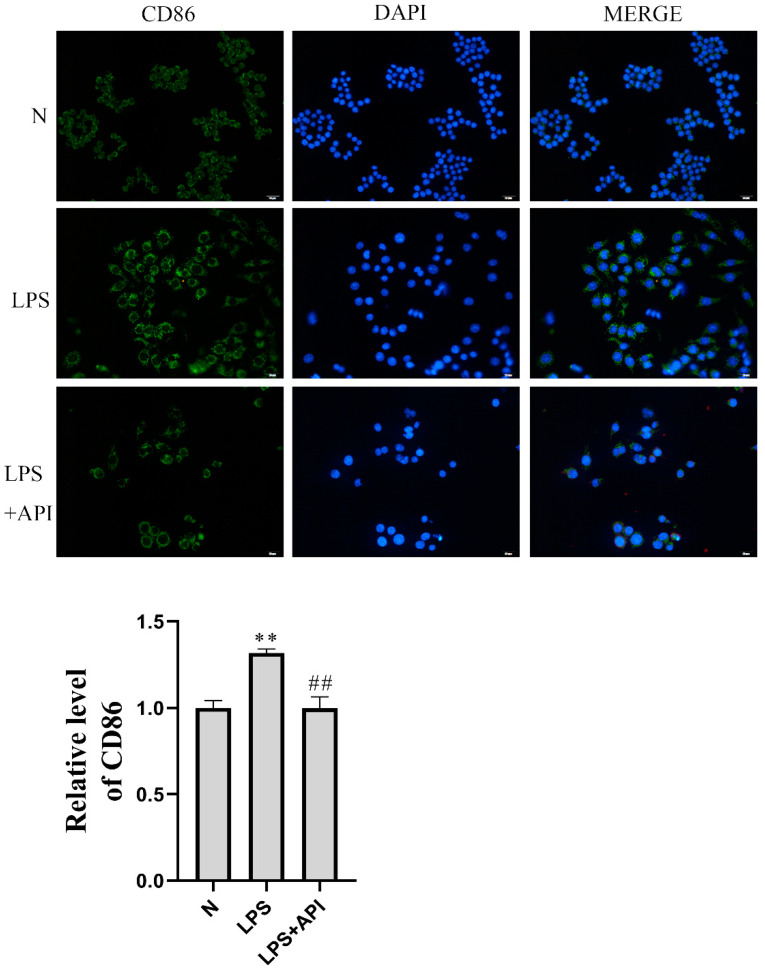
Apigenin inhibited macrophage M1 polarization. Above are representative immunofluorescence pictures of CD86 (magnification, × 200), The blue fluorescence is DAPI and the green fluorescence is CD86. Compared with group N, the median fluorescence intensity (MFI) of CD86 in the LPS group was significantly increased. Apigenin significantly reduced the expression level of the CD86 protein. The figure below shows the quantitative fluorescence intensity of CD86-positive cells: ** *p* < 0.01 versus normal group (N); ## *p* < 0.01 versus LPS group.

**Figure 5:**
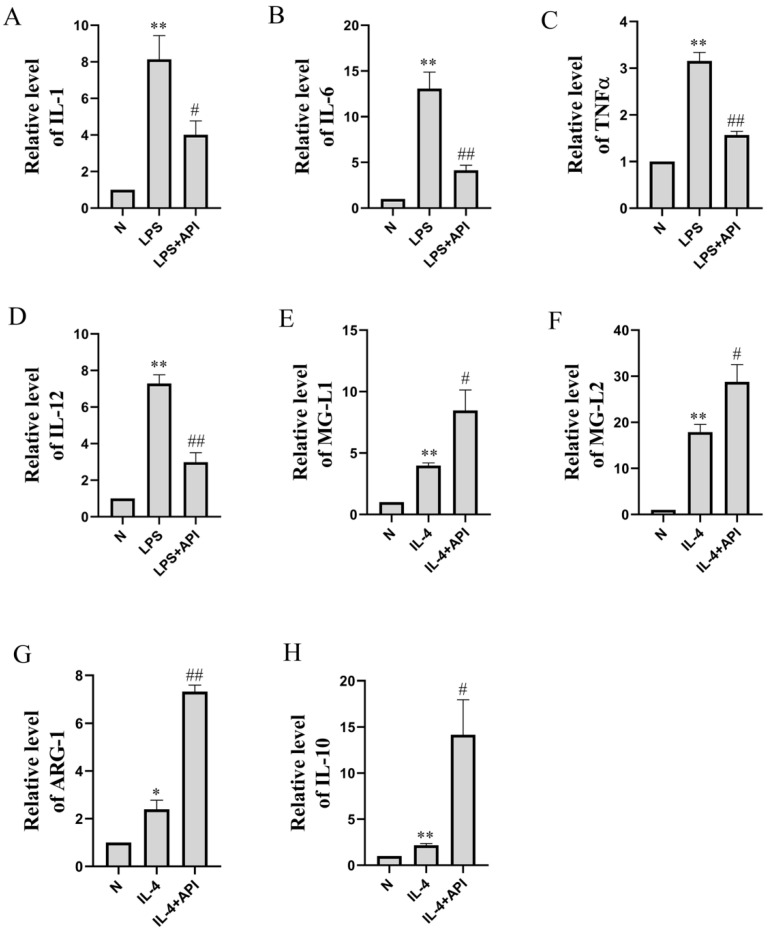
The effects of apigenin on the levels of inflammation-related mRNA of macrophages in the macrophage–chondrocyte coculture system. Relative quantities of inflammation-related genes are expressed as fold differences compared to the control. (**A**) IL-1, (**B**) IL-6, (**C**) TNF-α, (**D**) IL-12, (**E**) MG-L1, (**F**) MG-L2, (**G**) ARG-1, and (**H**) IL-10. Data are the mean ± SD of the mean (*n* = 3). * *p* < 0.05 versus normal group (N); ** *p* < 0.01 versus normal group (N); # *p* < 0.05 versus LPS or IL-4 group; ## *p* < 0.01 versus LPS or IL-4 group.

CD86 is a surface marker for M1 macrophages. As shown in Figure 4, the median fluorescence intensity (MFI) of CD86 increased significantly in the LPS-treated group of the macrophage–chondrocyte coculture system. Apigenin was then demonstrated to significantly reduce the levels of CD86 protein expression in the macrophage–chondrocyte coculture system. The qRT-PCR result also proved this (Figure 5A–D). In the macrophage–chondrocyte coculture system, the levels of M1 macrophage polarization markers, including IL-1 (*p* < 0.01), IL-6 (*p* < 0.01), TNFα (*p* < 0.01), and IL-12 (*p* < 0.01), were markedly elevated in the LPS group. Apigenin significantly inhibited the LPS-induced increase in IL-1 (*p* < 0.05), IL-6 (*p* < 0.01), TNFα (*p* < 0.01), and IL-12 (*p* < 0.01) levels. The above experimental results prove that apigenin inhibits LPS-induced macrophage M1 polarization in a macrophage–chondrocyte coculture system.

### 2.3. Apigenin Promotes Macrophage M2 Polarization in a Macrophage–Chondrocyte Coculture System

**Figure 6:**
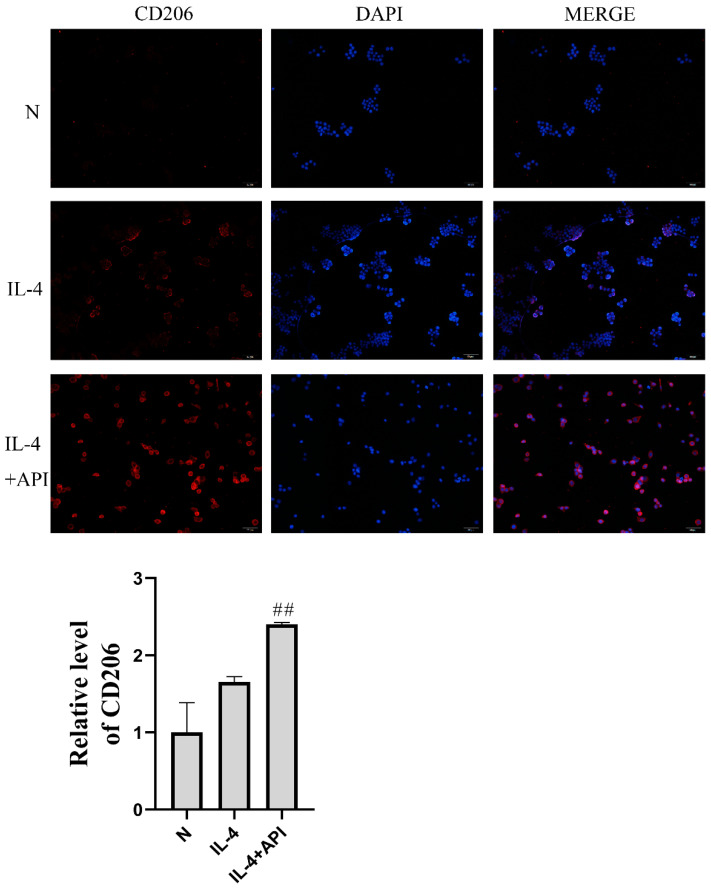
Apigenin promoted macrophage M2 polarization. Above are representative immunofluorescence pictures of CD206 (magnification, ×100), The blue fluorescence is DAPI and the red fluorescence is CD206. Compared with that in group N, the CD206 MFI in the IL-4 treatment group was significantly increased, and the CD206 level in the IL-4 + API group was further increased. The lower figure shows the quantitative fluorescence intensity of CD206-positive cells: ## *p* < 0.01 versus the IL-4 group.

To further explore the effects of apigenin on M2 polarization, RAW264.7 cells were treated with IL-4 to induce M2 polarization. As shown in Figure 6, the MFI of CD206 increased significantly in the IL-4-treated group of the macrophage–chondrocyte coculture system, and the level of CD206 increased further in the apigenin group. Additionally, PCR analysis showed the same trend as the immunofluorescence experiment. In the IL-4 group, MGL1 (Figure 5E, *p* < 0.01), MGL2 (Figure 5F, *p* < 0.01), ARG-1 (Figure 5G, *p* < 0.05), and IL-10 (Figure 5H, *p* < 0.01) were markedly elevated. After apigenin treatment, the levels of MGL1 (Figure 5E, *p* < 0.05), MGL2 (Figure 5F, *p* < 0.05), ARG-1 (Figure 5G, *p* < 0.01), and IL-10 (Figure 5H, *p* < 0.05) increased significantly during M2 polarization by IL-4. These results demonstrate that apigenin promoted M2 macrophage polarization in the macrophage–chondrocyte coculture system.

### 2.4. Apigenin Regulates Macrophage Polarization through the TRPM7-mTOR Pathway

**Figure 7:**
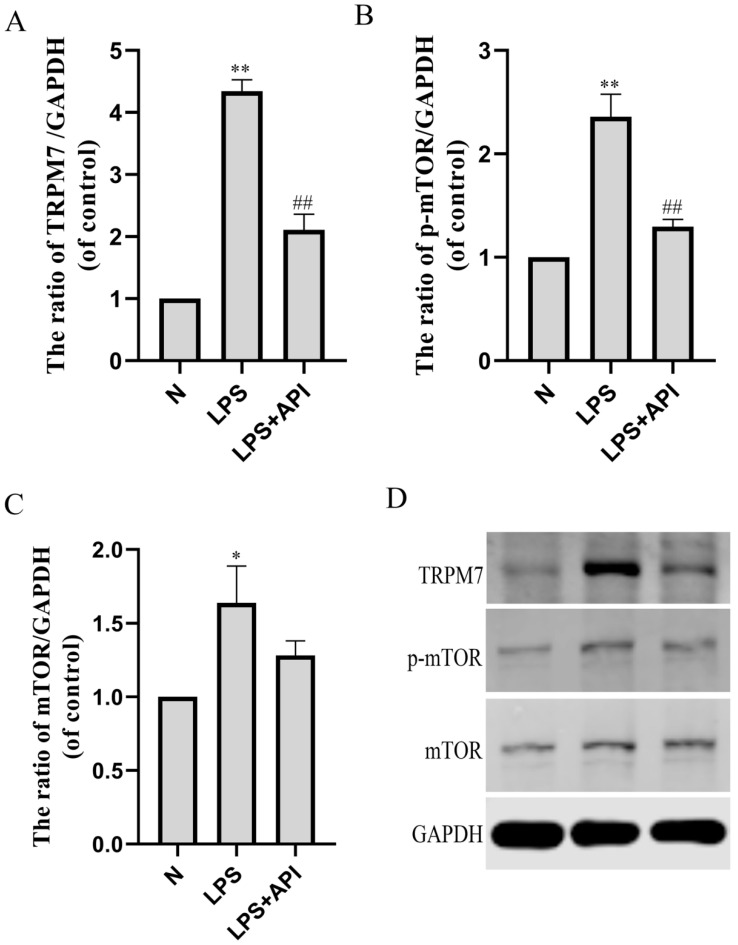
The effects of apigenin on the TRPM7, p-mTOR, and mTOR levels of macrophages in the macrophage–chondrocyte coculture system. (**A**) TRPM7 protein level; (**B**) p-mTOR protein level; (**C**) mTOR protein level; (**D**) Representative Western blotting images of TRPM7, p-mTOR, and mTOR. Data are the mean± SD of the mean. * *p* < 0.05 versus normal group (N); ** *p* < 0.01 versus normal group (N); ## *p* < 0.01 versus LPS group.

As shown in Figure 7, protein levels of TRPM7 (Figure 7A, *p* < 0.01) and mTOR (Figure 7C, *p* < 0.05), and the phosphorylation level of mTOR (Figure 7B, *p* < 0.01) were significantly increased during M1 polarization by LPS. Apigenin decreased the protein levels of TRPM7 (*p* < 0.01) and the phosphorylation level of mTOR (*p* < 0.01).

### 2.5. Apigenin Inhibits Chondrocyte Inflammation through the MAPK Pathway

**Figure 8:**
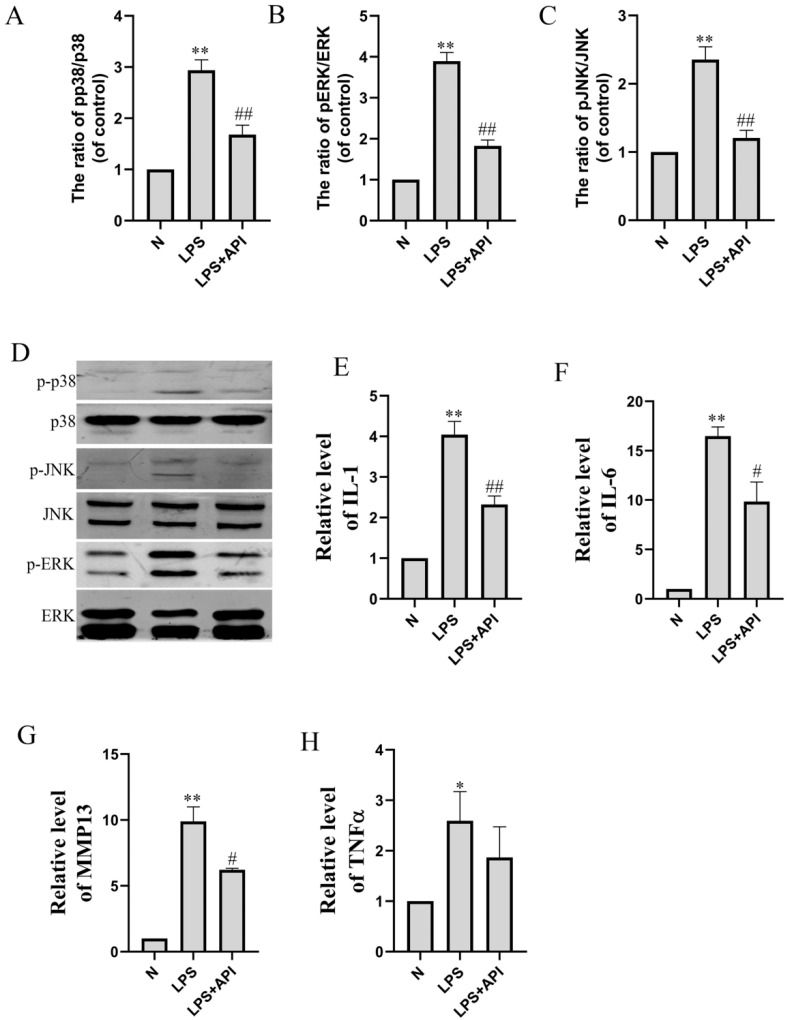
The effect of apigenin on the inflammation-related mRNA and protein levels of chondrocytes in the macrophage–chondrocyte coculture system. Relative quantities of inflammation-related genes are expressed as fold differences compared to the control. (**A**) p-p38/p38 protein level; (**B**) p-ERK/ERK protein level; (**C**) p-JNK/JNK protein level; (**D**) Representative Western blotting images of p-p38, p38, p-JNK, JNK, p-ERK, and ERK; (**E**) IL-1 mRNA level; (**F**) IL-6 mRNA level; (**G**) MMP13 mRNA level; (**H**) TNFα mRNA level. Data are the mean ± SD of the mean. * *p* < 0.05 versus normal group (N); ** *p* < 0.01 versus normal group (N); # *p* < 0.05 versus LPS group; ## *p* < 0.01 versus LPS group.

In the macrophage–chondrocyte coculture system, macrophage polarization induced by LPS can cause the inflammation of chondrocytes in the lower chamber of the Transwell plate. As shown in Figure 8, the mRNA levels of IL-1 (Figure 8E, *p* < 0.01), IL-6 (Figure 8F, *p* < 0.01), MMP13 (Figure 8G, *p* < 0.01), and TNFα (Figure 8H, *p* < 0.05) of chondrocytes in the lower chamber of the Transwell plate were significantly increased during macrophage polarization by LPS. Apigenin significantly inhibited the macrophage-polarization-induced increase in the IL-1 (*p* < 0.01), IL-6 (*p* < 0.05), and MMP13 (*p* < 0.05) levels of chondrocytes. As shown in Figure 8, apigenin decreased the phosphorylation level of p38 (Figure 8A, *p* < 0.01), JNK (Figure 8B, *p* < 0.01), and ERK (Figure 8C, *p* < 0.01). This implies that apigenin exerts its anti-inflammatory effects by blocking the MAPK pathway.

### 2.6. Apigenin Inhibits the Apoptosis of Chondrocytes Induced by Macrophage Polarization

**Figure 9:**
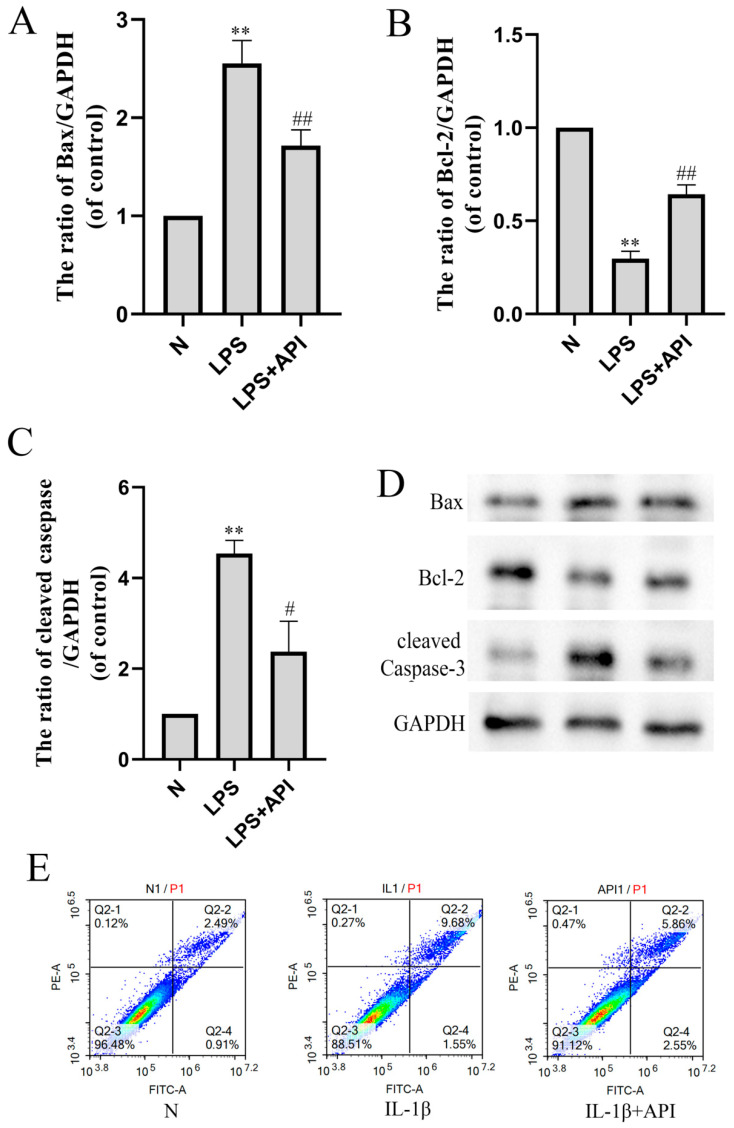
The effect of apigenin on chondrocyte apoptosis in the macrophage–chondrocyte coculture system. (**A**) Bax protein level; (**B**) Bcl-2 protein level; (**C**) cleaved-caspase3 protein level; (**D**) Representative Western blotting images of Bax, Bcl-2, and cleaved-caspase3; (**E**) Flow cytometry of chondrocytes in the macrophage–chondrocyte coculture model. In the two-color flow cytometry scatter plot, the Annexin V-FITC is represented on the X axis and the PI is represented on the Y axis. Living cells were double negative (Annexin V-FITC-/PI-), early apoptotic cells were Annexin V-FITC+/PI- positive (Annexin V-FITC+/PI-), late apoptotic cells were Annexin V-FITC and PI positive (Annexin V-FITC+/PI+). Data are the mean ± SD of the mean. ** *p* < 0.01 versus normal group (N); # *p* < 0.05 versus LPS group; ## *p* < 0.01 versus LPS group.

In our study, LPS (100 ng/mL) regulated the polarization of macrophages but could not directly induce the apoptosis of primary chondrocytes that were not cocultured with macrophages. However, in the macrophage–chondrocyte coculture system, chondrocyte cell apoptosis was observed in the lower chamber of the Transwell plate (Figure 9). In the previous result, we found that apigenin could inhibit chondrocyte apoptosis in OA mice.

The Western blot and flow cytometry analyses verified the results of an antiapoptotic effect of apigenin on OA mouse cartilage. As shown in Figure 9, protein levels of Bax (Figure 9A, *p* < 0.01) and cleaved-caspase3 (Figure 9C, *p* < 0.01) were significantly increased during M1 polarization by LPS in the macrophage–chondrocyte coculture system, while the protein level of Bcl-2 (Figure 9B, *p* < 0.01) was significantly decreased. Apigenin decreased the protein levels of Bax (Figure 9A, *p* < 0.01) and cleaved-caspase3 (Figure 9C, *p* < 0.01) and increased the protein level of Bcl-2 (Figure 9B, *p* < 0.05) significantly. In the two-color flow cytometry scatter plot, the Annexin V-FITC is represented on the X axis and the PI is represented on the Y axis. Living cells were double negative (Annexin V-FITC−/PI−), early apoptotic cells were Annexin V-FITCpositive (Annexin V-FITC+/PI−), late apoptotic cells were Annexin V-FITC and PI positive (Annexin V-FITC+/PI+). We can judge apoptosis by the percentage of cell population in the scatter plot. Compared with the N group, the apoptosis of chondrocytes increased after IL-1β induction, and apigenin reduced the apoptosis of chondrocytes.

## 3. Discussion

OA is a degenerative disease of articular cartilage with high incidence, affecting more than 500 million people worldwide (~7% of the global population), and is induced by various factors in the elderly (>65 years of age) [20]. OA not only affects the articular cartilage but also involves pathological changes across all the joint tissues, including subchondral bone, ligaments, capsules, synovium, and periarticular muscles, as a result of a combination of risk factors [21]. Multiple studies have documented that low-grade synovial inflammation (synovitis) contributes to cartilage degeneration and pain progression in OA, although the etiology of OA is complicated [20,22,23]. During the process of OA, macrophages can become activated M1 macrophages that can release inflammatory mediators and MMPs [23].

Recent studies have shown that a variety of flavonoids can act on osteoarthritis and delay its progression. Licochalcone A (Lico A) inhibits the NLRP1 inflammatorome through the nuclear factor erythropoie-3 associated factor 2 (Nrf2)–heme oxygenase-2 (HO-1)–nuclear factor κ-B (NF-κB) axis. Nrf2 small interfering RNA (siRNA) can reverse the antipyroptosis effect of Lico A in mouse OA chondrocytes and has a potential therapeutic effect on OA [24]. Quercetin inhibits ER stress by activating the sirtuin1–adenosine monophosphate activated protein kinase (SIRT1–AMPK) signaling pathway. Protective effects of quercetin were also observed in rat models of OA [25]. Luteolin decreased the IL-1β-induced production of NO, PGE2, TNF-α, MMP-2, MMP-8, and MMP-9 and the expression of COX-2, iNOS, MMP-1, MMP-3, and MMP-13, and it reversed the IL-1β-induced degradation of collagen II [26]. Tangeretin can eliminate OA progression by inhibiting inflammation and ECM degradation in chondrocytes and animal models through the Nrf2–NF-κB and MAPK–NF-κB pathways [27]. Icariin has a strong inhibitory effect on proinflammatory signaling, such as NF-κB and MAPK, and can also upregulate anti-inflammatory signaling, such as GR and Nrf2 [28]. Fisetin is available for orthopedic biologic therapy as an adjunct to orthopedic surgery to improve clinical outcomes [29].

In this study, apigenin protected chondrocytes by repressing M1 macrophage polarization and promoting M2 macrophage polarization. Transient receptor potentioid melatonin seven (TRPM7) is a nonselective Ca^2+^ conducting ion channel that plays an important role in lipopolysaccharide (LPS)-induced activation of mouse macrophages [30]. mTOR plays an important role in the regulation of the polarization of macrophages. Previous studies have confirmed that the activation of mTORC1 can inhibit autophagy and promote the apoptosis of articular chondrocytes during OA development [31]. Apigenin can attenuate inflammatory responses in a macrophage–chondrocyte coculture system induced by macrophage polarization through the TRPM7–mTOR pathway. In this study, we demonstrated the anti-inflammatory and antiapoptotic effects of apigenin in a macrophage–chondrocyte coculture system. Mitogen-activated protein kinases (MAPKs) are crucial regulators of cellular pathology and physiology, playing a crucial role in chondrogenic differentiation, including JNK, ERK, and p38 [32]. We found that apigenin inhibited chondrocyte apoptosis and inflammation through the MAPK pathway. Apigenin is a well-known nutraceutical compound that has been shown to have effects on diabetes, amnesia and Alzheimer’s disease, depression, insomnia, cancer, and female diseases [12,33]. It has also been demonstrated that apigenin modulates bone formation by regulating AnxA6 and TNAP [34]. We demonstrated herein that the chondroprotective effect of apigenin is mainly ascribed to its regulatory effects on macrophage polarization. There is increasing evidence that synovial inflammation plays a major role in the progression of osteoarthritis. Therefore, alleviating synovial inflammation may be an effective method of treating patients with osteoarthritis [35,36].

## 4. Materials and Methods

### 4.1. Reagents

Apigenin (98% in purity) was obtained from Aladdin (Shanghai, China). GAPDH (AP0063) was purchased from Bioworld (Nanjing, China). IL-1 beta (26048-1-AP), IL-6 (66146-1-lg), MMP3 (17873-1-AP), and MMP13 (18165-1-AP) were purchased from Proteintech (Wuhan, China). JNK (ET1601-28), P-JNK (ET1609-42), ERK (ET1601-29), P-ERK (ET1610-13), P38 (ET1602-26), P-P38 (ER2001-52), TRPM7 (ER61334), p-mTOR (HA600094), and mTOR (ET1608-5) were purchased from HUABIO (Hangzhou China). Bax (#40635) and Bcl-2 (#40639) were purchased from SAB (Nanjing, China). Cleaved-caspase3 (TA7022S) was purchased from Abmart (Shanghai, China).

### 4.2. Cell Culture of Chondrocytes and Macrophages

The isolation and identification of primary chondrocytes by Alcian blue staining and immunocytochemical staining of type II collagen from mice were performed as previously described [37]. In brief, the primary chondrocytes used for experiments were prepared from the knee joint cartilage of newborn mice. After digestion by trypsinase and collagenase II, sequentially, the cells were maintained in DMEM/F12 1:1 + 10% fetal bovine serum. The cells identified as positive for Alcian blue staining and immunocytochemical staining of type II collagen were used in subsequent experiments. RAW264.7 was purchased from the Shanghai Institute of Cell Biology (Shanghai, China). High-glucose DMEM containing 10% FBS was used for RAW264.7 cell culturing. The cells were incubated in a 37 °C incubator with 5% CO_2_ [38].

Transwell coculture systems were performed. Chondrocytes were seeded onto 12-well culture plates at 1 × 10^6^ cells per 1 mL. After overnight seeding, the cells were washed 3 times with PBS, and RAW cells were directly added to the chondrocytes. The control group received serum-free medium only. After apigenin treatment, the supernatant and cells were collected separately for further analyses [39]. M1 macrophages were polarized by the addition of LPS (100 ng/mL) and IFN-γ (50 ng/mL) for 24 h, and M2 macrophages were obtained by the addition of IL-4 (20 ng/mL) for 24 h [40]. The cells of the drug-treated group were treated with apigenin (10 μM). Then, the polarization transitions were evaluated via q-PCR, Western blot, and immunohistochemical staining.

### 4.3. Western Blot Analysis

Protein expression was measured via Western blot analysis. In brief, cells were washed with PBS and lysed using a RIPA buffer with 1% phenylmethanesulfonyl fluoride (PMSF); then, the protein concentration of cell lysates was quantified using the BCA method (Beyotime, Shanghai, China). Subsequently, 20 μg amounts of protein were resolved on sodium dodecyl sulfate–polyacrylamide gel electrophoresis (SDS-PAGE) 10% gel and transferred onto a polyvinylidene fluoride (PVDF) membrane. After transfer, membranes were blocked with 5% bovine serum album in Tris-buffered saline at room temperature before incubation overnight with primary antibodies in a blocking buffer at 4 °C. The excess primary antibody was removed by washing with Tris-buffered Saline and 0.1% Tween-20 (TBST) three times for 5 min, and the membranes were subsequently incubated with secondary antibodies at room temperature for 1 h. After rinsing with TBST, the band density was quantified using ImageJ software.

### 4.4. qRT-PCR

Macrophages were inoculated in the upper compartment and chondrocytes were inoculated in the lower compartment of the Transwell plate. The cells were cultured for another 48 h after administration before they were harvested. The methods and parameters of the qPCR assay were employed as previously described. Total RNA was extracted from collected cells at the end of the treatment period using Trizol reagent (cat#: 9109; Invitrogen, Carlsbad, CA, USA) and was reverse transcribed into cDNA using the PrimeScript™ RT Reagent Kit (cat#: RR037A; TAKARA BIO INC, Shiga, Japan) according to the manufacturer’s instructions. The primer sequences used for qPCR are listed in Table 1. The cycling conditions were 95 °C for 10 min, followed by 40 cycles of 95 °C for 15 s and 60 °C for 1 min. The relative graphs and statistical analyses of transcript quantities were calculated using the 2^−ΔΔCt^ method with GAPDH as the endogenous reference gene amplified from the samples. The q-PCR analysis was carried out 3 times.

### 4.5. Immunofluorescent Staining

Macrophages were stained for immunofluorescence on coverslips according to the standard protocol. Following removal of the supernatant, cells were fixed in a fixation/permeabilization buffer for 30 min at 4 °C. Next, the cells were incubated with primary antibodies against CD86 or CD206 overnight at 4 °C. After that, the cells were rinsed three times in PBS, then incubated with FITC-conjugated secondary antibodies. The coverslips of the three groups were counterstained with DAPI, washed three times in PBS, and imaged under a fluorescent microscope [41].

### 4.6. Flow Cytometry

The apoptotic degree of chondrocytes in each group was measured by flow cytometry using the Annexin V-FITC/PI apoptosis detection kit according to the manufacturer’s instructions (cat#: A211-01; Vazyme, Nanjing, China). Flow cytometry analysis was performed using the NovoExpress (Agilent Technologies, Inc., Nanjing, China).

### 4.7. OA Protocol

The animal experimental operations were approved by the Institutional Animal Care and Use Committee of Xuzhou Medical University (202209S020). Modified Hulth surgery was performed in mice to induce experimental OA. C57BL/6J mice (8 weeks of age) were used for this study. In the treatment study, modified Hulth surgery in the right knee joints was performed in mice to induce experimental OA according to previous studies. The nonoperated right knees were used as ipsilateral controls. OA was surgically induced by modified Hulth (the surgical modified Hulth model of osteoarthritis in the 129/SvEv mouse). Mice were divided into 3 groups (6 mice per group) by random assignment: sham, modified Hulth, and modified Hulth + apigenin. Animals were gavaged daily for 4 weeks. Then, the mice were sacrificed to evaluate OA severity and joint inflammation.

### 4.8. Histological and Immunohistochemical Analyses

The femoral cartilage tissue from mice was fixed in 10% formalin, decalcified, and embedded in paraffin. Five-micrometer serial sections were obtained and stained with hematoxylin and eosin (H&E), Safranin-O Fast green, and toluidine blue. In order to detect the apoptosis of chondrocytes, terminal deoxynucleotidyl transferase-mediated dUTP nick-end labeling (TUNEL) staining was performed using a TUNEL assay kit according to the manufacturer’s instructions (Beyotime, Shanghai, China). Staining was visualized by fluorescence microscopy.

### 4.9. Statistical Analyses

Statistical analyses were performed using SPSS software, and results are presented as the mean ± SD. Graphs were drawn using GraphPad Prism (version 6.0 for Windows). Every experiment was repeated at least three times. A *p*-value of 0.05 or less was considered statistically significant.

## 5. Conclusions

Apigenin could ameliorate modified Hulth-induced cartilage damage in OA mice. Apigenin attenuated inflammatory responses and alleviated chondrocyte apoptosis induced by macrophage polarization in a macrophage–chondrocyte coculture system through the TRPM7–mTOR pathway. These results suggest a potential therapeutic treatment strategy involving apigenin for OA.

## Figures and Tables

**Table 1:** Primer sequences used in qRT-PCR.

	Sequence (5′ to 3′)
IL-1	F: CTG CAC TAC AGG CTC CGA
	R: GCC ACA GGT ATT TTG TCG TT
IL-6	F: TTA GCC ACT CCT TCT GTG ACT CC
	R: ACC CCA ATT TCC AAT GCT CT
IL-12a	F: GAC CTG TTT ACC ACT GGA ACT A
	R: GAT CTG CTG ATG GTT GTG ATT C
TNF-α	F: TCG TAT GAA ATG GCA AAT CG
	R: GGT CCC AAC AAG GAG GAG
MGL1	F:TGC AAC AGC TGA GGA AGG ACT TG
	R:AAC CAA TAG CAG CTG CCT TCA TGC
MGL2	F:GCA TGA AGG CAG CTG CTA TTG GTT
	R:TAG GCC CAT CCA GCT AAG CAC ATT
ARG-1	F:CAT ATC TGC CAA AGA CAT CGT G
	R:GAC ATC AAA GCT CAG GTG AAT C
IL-10	F:AGG CGC TGT CAT CGA TTT CT
	R:TGG AGT CCA GCA GAC TCA AT
MMP3	F:GGA GGC AGC AGA GAA CCT AC
	R:TCC AAC CCG AGG AAC TTC TG
MMP13	F:CAG TGC TGC GGT TCA CTT TG
	R:TCA TCA TAA CTC CAC ACG TGG TT

## Data Availability

The data presented in this study are available on request from the corresponding author.

## Sample Availability

Samples of the compounds are not available from the authors.
